# Análise do desempenho dos hospitais públicos e privados que atendem ao Sistema Único de Saúde

**DOI:** 10.1590/0102-311XPT156023

**Published:** 2024-11-11

**Authors:** Leandro Manassi Panitz, David Nadler Prata, Waldecy Rodrigues

**Affiliations:** 1 Ministério da Saúde, Brasília, Brasil.; 2 Universidade Federal do Tocantins, Palmas, Brasil.

**Keywords:** Sistemas de Informação Hospitalar, Assistência Hospitalar, Benchmarking, Sistema Único de Saúde, Hospital Information Systems, Hospital Care, Benchmarking, Unified Health System, Sistemas de Información Hospitalaria, Atención Hospitalaria, Benchmarking, Sistema Único de Salud

## Abstract

O artigo tem como objetivo analisar o desempenho da rede hospitalar do Sistema Único de Saúde (SUS) com base nos bancos de dados nacionais do Sistema de Informações Hospitalares (SIH) e do Cadastro Nacional de Estabelecimentos de Saúde (CNES). A pesquisa utilizou um conjunto de indicadores abrangendo a produção de internações, o perfil de atendimentos, a qualidade e o desempenho, associados ao porte dos hospitais e à natureza jurídica. Para análise de dados, empregou-se: a análise da variância com teste de Tukey-Kramer para evidenciar as diferenças entre hospitais públicos e privados; a análise de moderação para verificar o efeito do porte hospitalar; e o modelo Pabon Lasso para integrar os resultados. Estes demonstram que o aumento no número de leitos influencia o desempenho dos indicadores de maneira distinta para hospitais públicos e privados. Hospitais públicos apresentaram ganhos de eficiência de escala superiores com o aumento de leitos e os privados sem fins lucrativos, um desempenho superior no conjunto de indicadores e mais equilibrado nos diferentes portes. A aplicação do modelo Pabon Lasso demonstrou que hospitais pequenos, tanto públicos quanto privados, apresentam baixo desempenho, e evidenciou também que, a partir do médio porte, os hospitais públicos e privados apresentam um bom desempenho. No entanto, cada categoria exibe particularidades em seu perfil de performance, refletindo uma diversidade de práticas e resultados operacionais. Desse modo, o estudo confirma achados anteriores de que o desempenho hospitalar tende a melhorar com o aumento do número de leitos, mas revela também que ele varia significativamente em função da natureza jurídica dessas instituições.

## Abstract (en)

This article aims to analyze the performance of the Brazilian Unified National Health System (SUS, acronym in Portuguese) hospital network based on the national databases of the Brazilian Hospital Information System (SIH, acronym in Portuguese) and the Brazilian National Registry of Health Establishments (CNES, acronym in Portuguese). The research used a set of indicators covering the production of admissions, the profile, quality and performance of care, associated with hospital size and legal nature. For data analysis, the Analysis of Variance with Tukey-Kramer test was used to highlight the differences between public and private hospitals, the moderation analysis to verify the effect of hospital size and the Pabon Lasso model to integrate the results. The results show that the increase in the number of beds influences the performance of the indicators differently for public and private hospitals. Public hospitals showed higher gains in scale efficiency with the increase in beds, whereas private non-profit hospitals showed a higher performance in the set of indicators and a more balanced performance in the different sizes. The application of the Pabon Lasso model showed that small hospitals, both public and private, perform poorly. It also showed that from medium-sized onwards, public and private hospitals perform well. However, each category displays particularities in its performance profile, reflecting a diversity of practices and operational results. This study thus confirms previous findings that hospital performance tends to improve as the number of beds increases, but also reveals that it varies significantly depending on the legal nature of these institutions.

## Resumen

Este artículo tiene como objetivo analizar el desempeño de la red hospitalaria del Sistema Único de Salud brasileño (SUS, por sus siglas en portugués) a partir de las bases de datos nacionales del Sistema de Información Hospitalaria (SIH, por sus siglas en portugués) y del Registro Nacional de Establecimientos de Salud (CNES, por sus siglas en portugués). Para ello, se utilizó un conjunto de indicadores que cubren la producción de hospitalizaciones, el perfil de la atención, la calidad y el desempeño asociados con la capacidad de los hospitales y la naturaleza legal. El análisis de varianza con prueba de Tukey-Kramer se utilizó en el análisis de datos para identificar las diferencias entre los hospitales públicos y privados; el análisis de moderación para verificar el efecto de la capacidad del hospital; y el modelo Pabon Lasso para integrar los resultados. Los resultados mostraron que un aumento en la cantidad de camas influye en el desempeño de los indicadores de manera distinta tanto en los hospitales públicos como en los privados. Los hospitales públicos tuvieron mayor ganancia de eficiencia de escala con el aumento de camas, mientras que los hospitales privados sin fines de lucro mostraron un mejor desempeño en el conjunto de indicadores y más equilibrado en las diferentes capacidades. La aplicación del modelo Pabon Lasso demostró que los pequeños hospitales, tanto públicos como privados, tuvieron un bajo desempeño. También mostró que los hospitales públicos o privados de mediano tamaño tuvieron un buen desempeño. Sin embargo, cada categoría guarda particularidades en su perfil de desempeño, reflejando una diversidad de prácticas y resultados operativos. Por lo tanto, los resultados de este estudio confirman los hallazgos previos de que el desempeño hospitalario tiende a mejorar con el aumento del número de camas, pero también revela que hay una variación significativa dependiendo de la naturaleza legal de estas instituciones.

## Introdução

A Saúde Pública no Brasil enfrenta inúmeros desafios que impactam diretamente no acesso e na qualidade dos serviços oferecidos à população. Entre eles, a gestão eficiente na alocação de recursos públicos se apresenta com uma questão central e complexa para um sistema de saúde que opera historicamente com um significativo subfinanciamento ^1^.

Entre as diversas áreas de atuação dos sistemas de saúde, a atenção à saúde é uma das mais críticas e financeiramente exigentes, abrangendo uma ampla gama de serviços que vão desde os básicos até os altamente especializados, e uma prestação intensa de serviços ambulatoriais e hospitalares à população. Os serviços de saúde realizados pelas unidades hospitalares correspondem ao nível de atenção terciário e são caracterizados pelo uso dos mais altos níveis de complexidade e densidade tecnológica, resultando em altos custos financeiros.

A avaliação do desempenho hospitalar constitui um componente crucial na gestão de sistemas de saúde, particularmente em contextos de recursos limitados e crescentes demandas de eficiência e qualidade. Nesse sentido, a importância dos indicadores de saúde reside na sua capacidade de monitorar e melhorar a qualidade e o desempenho dos serviços de saúde ^2^. A análise de desempenho hospitalar pode ser realizada a partir de diferentes perspectivas e pode englobar múltiplas dimensões como eficiência, segurança e foco no paciente, orientação do profissional de saúde e governança ^3^. No entanto, a disponibilidade limitada de dados sobre as atividades hospitalares resulta em medições difíceis, e por isso os estudos sobre esse tema devem explorar a possibilidade de construção de indicadores a partir de informações oficiais disponíveis ^4^. Disso resultam estudos de análise de desempenho que costumam focar na análise de variáveis relacionadas ao movimento do paciente e à utilização dos leitos hospitalares ^3^.

Entre os métodos empregados para a avaliação do desempenho hospitalar, destacamos a importância dos três utilizados neste estudo. A análise de variância (ANOVA), para evidenciar as diferenças entre as médias de grupos de indicadores e determinar se existem variações estatisticamente significativas entre os grupos de hospitais. A análise de moderação, que permite identificar variáveis moderadoras que impactam a intensidade ou a direção das relações estabelecidas entre variáveis dependentes e independentes podendo ser utilizada para compreender como fatores adicionais influenciam as dinâmicas de desempenho hospitalar. E o modelo Pabon Lasso como modelo gráfico para a análise comparativa do desempenho de hospitais com base em três indicadores de desempenho tradicionais ^5^, permitindo identificar áreas de eficiência e ineficiência dentro de um conjunto de hospitais.

Os estudos de desempenho hospitalar são particularmente relevantes no contexto brasileiro pois há uma participação muito expressiva da esfera privada na prestação de serviços hospitalares ao Sistema Único de Saúde (SUS). Conforme Paim et al. ^6^, o sistema de saúde brasileiro é constituído por uma rede complexa de prestadores públicos e privados estabelecidos em diferentes períodos históricos, e no qual atualmente o subsistema privado de saúde se imbrica com o setor público oferecendo serviços terceirizados pelo SUS.

Deste modo, o objetivo do estudo foi apresentar uma análise do desempenho dos hospitais públicos e privados que prestam serviços ao SUS. A questão central que a pesquisa busca responder é se a natureza jurídica e o porte hospitalar influenciam no desempenho dos hospitais em análise.

## Métodos

Este estudo transversal analisou os dados das unidades hospitalares brasileiras que prestaram serviços ao SUS no ano de 2019, com base nos bancos de dados oficiais e de domínio público disponibilizados pelo Ministério da Saúde. As informações sobre as características e a estrutura dos estabelecimentos de saúde foram obtidas do Cadastro Nacional de Estabelecimentos de Saúde (CNES), e as informações sobre internações hospitalares foram obtidas do banco de dados do Sistema de Informações Hospitalares (SIH).

O estudo partiu de uma base de dados com um total de 8.444 estabelecimentos de saúde com leitos cadastrados no CNES em 2019, dos quais foram selecionados apenas os 5.930 com leitos hospitalares disponíveis para o SUS. Adicionalmente, foram aplicados três critérios de seleção: (1) possuir internações hospitalares registradas no SIH durante o ano de 2019; (2) ter cinco ou mais leitos disponíveis para o SUS, fundamentado nos critérios mínimos de leitos da *Portaria GM/MS nº 1044,* de 2004, e considerando que as unidades com menos de cinco leitos apresentaram baixa regularidade de produção e número pouco significativo de internações; e (3) ser de uma tipologia de estabelecimento de saúde tipicamente hospitalar (hospital geral, hospital especializado, pronto-socorro geral, pronto-socorro especializado ou unidade mista), sendo essa última também chamada de hospital-unidade sanitária com características híbridas de posto de saúde e hospital ^7^.

A aplicação dos critérios de seleção resultou em um universo de pesquisa de 4.449 unidades hospitalares, eliminando 415 estabelecimentos por terem menos de cinco leitos disponíveis para o SUS, 435 estabelecimentos por não serem instituições tipicamente hospitalares e 631 por falta de produção de Autorizações de Internação Hospitalar (AIH) em 2019.

As informações do banco de dados do CNES possibilitaram identificar atributos inerentes às unidades hospitalares (Quadro 1) que foram considerados essenciais para caracterizar essas instituições em relação ao seu porte, tipologia, regime jurídico, e inserção no território nacional.

**Quadro 1:** Lista e descrição dos atributos dos hospitais.

ATRIBUTOS	DESCRIÇÃO	CLASSIFICAÇÃO
Natureza jurídica	Classificação definida pelo CONCLA, que identifica a organização jurídica e administrativa das instituições e sua relação com o sistema legal do país. Foi utilizado o nível mais agregado da nomenclatura para classificar os hospitais: (1) administração pública; (2) entidades empresariais; e (3) entidades sem fins lucrativos.	Hospitais públicos Hospitais empresariais Hospitais sem fins lucrativos
Porte hospitalar	Classificação de porte hospitalar elaborada com base no número de leitos hospitalares cadastrados no CNES, baseada na categorização de Cherubin ^32^ e com estratificação adicional do pequeno porte fundamentada na Política Nacional para os Hospitais de Pequeno Porte ^20^ e na Classificação adotada pelos estudos do PROADESS ^33^.	Pequeno I (5 a 30) Pequeno II (31 a 50) Médio (51 a 150) Grande (151 a 500) Especial (acima de 500)
Tipo de hospital	Baseado na tipologia de estabelecimentos de saúde definida pelo CNES com base em critérios como o tipo de atendimento oferecido, a complexidade dos serviços prestados e a estrutura física disponível. Foram selecionados somente os tipos como atividade de internação hospitalar.	05 - Hospital Geral 07 - Hospital Especializado 15 - Unidade Mista 20 - Pronto Socorro Geral 21 - Pronto Socorro Especializado
Regiões brasileiras	Divisão regional do Brasil definida pelo IBGE como o agrupamento de estados com características geográficas, sociais e econômicas semelhantes.	Norte Nordeste Centro-oeste Sudeste Sul

CNES: Cadastro Nacional de Estabelecimentos de Saúde; CONCLA: Comitê Nacional de Classificação; IBGE: Instituto Brasileiro de Geografia e Estatística; PROADESS: Projeto Avaliação do Desempenho do Sistema Saúde.

A combinação das informações do CNES e SIH possibilitaram o cálculo dos 12 indicadores apresentados na Quadro 2, considerados relevantes para abordar o perfil de atendimento e o desempenho das unidades hospitalares.

**Quadro 2:** Lista e descrição dos indicadores hospitalares calculados.

INDICADORES	DESCRIÇÃO
Produção	Aspectos físico-financeiros das AIH
Valor por internação	Valor financeiro apresentado nas AIH
Valor por dia de internação	Valor financeiro dividido pelos dias de permanência das AIH
Volume de internações	Quantidade de AIH
Perfil de atendimento	Características macro do perfil de atendimentos realizados pelos hospitais
Internações por ICSAP	Proporção de internações por ICSAP pelo total de internações
Internações de alta complexidade	Proporção de internações de alta complexidade pelo total de internações
Internações de urgência	Proporção de internações de urgência pelo total de internações
Internações de não residentes	Proporção de internações de não residentes no município do estabelecimento de saúde pelo total de internações
Qualidade	Dimensões da resolubilidade dos serviços prestados
Taxa de mortalidade hospitalar	Proporção de internações com desfecho em óbito pelo total de internações
Taxa de transferência hospitalar	Proporção de internação com desfecho de transferência pelo total de internações
Desempenho	Indicadores utilizados em estudos para medir o desempenho das instituições hospitalares
Tempo médio de permanência	Média de dias de permanência em internação
Índice de rotatividade	Razão entre o número de internações com desfecho de alta e óbito pelo total de leitos disponíveis ao SUS
Taxa de ocupação hospitalar	Razão entre número total de dias de internação das AIH pelo total de dias disponíveis para internação nos leitos SUS

AIH: Autorizações de Internação Hospitalar; ICSAP: internações por condições sensíveis à atenção primária; SUS: Sistema Único de Saúde.

Na fase exploratória do estudo, foi realizada uma análise do comportamento dos 12 indicadores hospitalares em relação aos atributos das unidades hospitalares por meio da ANOVA. Este estudo utilizou o Welch’s ANOVA pela sua capacidade de fornecer resultados mais confiáveis ao considerar a suposição de homogeneidade de variâncias, em conjunto com o teste de amplitude múltipla de Tukey-Kramer para comparações *post hoc*.

Na primeira etapa de análise de dados, os resultados da análise de variância com foco nas diferentes naturezas jurídicas dos hospitais foram demonstrados por meio da Tabela 1, com indicadores expressos em média, desvio padrão e em valores dos testes estatísticos.

**Tabela 1:** Indicadores hospitalares por natureza jurídica dos hospitais.

Indicadores hospitalares	Hospitais públicos (n = 2.505)	Hospitais sem fins lucrativos (n = 1.601)	Hospitais empresariais (n = 343)	Todos os hospitais (N = 4.449)	ANOVA		Tukey-Kramer		
							Públicos-Sem fins lucrativos	Empresariais-Sem fins lucrativos	Públicos-Empresariais
	Média	Média	Média	Média	F	Valor de p	Valor de p	Valor de p	Valor de p
Produção									
Valor por internação (R$)	615,88	1.020,54	2.204,47	883,97	77,44	< 0,0001	< 0,0001	< 0,0001	< 0,0001
Valor por dia de internação (R$)	115,33	191,00	487,77	171,28	106,57	< 0,0001	< 0,0001	< 0,0001	< 0,0001
Volume de internações (R$)	210,45	271,46	174,99	229,67	16,49	< 0,0001	< 0,0001	< 0,0001	0,1996
Perfil de atendimento									
Internações por ICSAP (%)	31,41	26,09	25,39	29,03	41,85	< 0,0001	< 0,0001	0.8214	< 0,0001
Internações de alta complexidade (%)	1,42	5,00	15,14	3,77	81,35	< 0,0001	< 0,0001	< 0,0001	< 0,0001
Internações de urgência (%)	85,02	79,78	72,71	82,18	31,95	< 0,0001	< 0,0001	< 0,0001	< 0,0001
Internações de não residentes (%)	18,28	26,27	34,57	22,41	103,51	< 0,0001	< 0,0001	< 0,0001	< 0,0001
Qualidade									
Taxa de mortalidade hospitalar (%)	3,10	4,01	2,77	3,40	29,83	< 0,0001	< 0,0001	< 0,0001	0,3425
Taxa de transferência hospitalar (%)	6,00	4,00	3,00	5,00	46,37	< 0,0001	< 0,0001	0,0027	< 0,0001
Desempenho									
Tempo médio de permanência	5,59	5,97	6,21	5,78	4,75	0,0088	0,0184	0,6270	0,0372
Índice de rotatividade	2,34	2,99	2,60	2,60	55,16	< 0,0001	< 0,0001	0,0015	0,0554
Taxa de ocupação hospitalar (%)	43,74	54,37	42,97	47,51	61,48	< 0,0001	0,9084	< 0,0001	< 0,0001

Na segunda etapa de análise de dados, o desempenho dos hospitais públicos e privados foi analisado por meio dos três indicadores de desempenho em conjunto com o porte hospitalar, considerados como as principais categorias para uma análise de moderação ^8^ a fim de verificar possíveis efeitos da natureza jurídica nessa relação.

Neste contexto, temos que: *Y* denota o valor previsto do indicador de desempenho médio para um hospital específico; *X* simboliza o número de leitos, servindo como *proxy* para o tamanho do hospital (i.e., número de leitos); *Z* representa a natureza jurídica do hospital (i.e., empresarial *vs.* sem fins lucrativos *vs.* público); o termo *XZ* encapsula a interação entre o número de leitos e a natureza jurídica; e os coeficientes *β* são parâmetros que indicam o peso de cada componente na equação. A relação moderadora é, portanto, expressa como:

$$\overset{-}{Y}=\beta_{0}+\beta_{1}Χ+\beta_{2}Z+\beta_{3}ΧΖ$$

Na última etapa de análise de dados, aplicou-se o modelo Pabon Lasso ^5^, que permitiu a construção de uma representação gráfica do desempenho dos hospitais, integrando os três indicadores de desempenho em relação à natureza jurídica e o porte de leitos.

O processamento de dados e as análises estatísticas na base de dados da pesquisa foram realizados utilizando o software IBM SPSS Statistics, versão 26 (https://www.ibm.com/).

## Resultados

Os serviços hospitalares realizados no âmbito do SUS se caracterizam por uma oferta bem equilibrada entre a esfera pública e privada. As instituições públicas representam 56,3% dos hospitais brasileiros e 53,5% do total de leitos disponíveis ao SUS. Já em relação às instituições privadas, há uma participação muito significativa de hospitais sem fins lucrativos, que somam 36% dos hospitais e 40% dos leitos disponíveis ao SUS. Os hospitais empresariais representam apenas 7,7% dos hospitais e 6,7% dos leitos SUS.

Os 4.449 hospitais brasileiros do estudo estão presentes em 2.992 municípios, abarcando cerca de 54% dos municípios do país e evidenciando uma rede hospitalar abrangente e amplamente difundida no território nacional. A maioria dos hospitais estão concentrados nas regiões Nordeste (33,6%) e Sudeste (29,9%), com 16,9% na Região Sul, 10,1% no Centro-oeste e 9,5% no Norte do país.

Os resultados obtidos na pesquisa evidenciam uma ampla predominância de hospitais de pequeno porte (até 50 leitos) com grande capilaridade no território nacional, representando cerca de 61% dos hospitais brasileiros e presentes em 48% dos municípios brasileiros. Hospitais públicos representam 60% dos hospitais de pequeno porte, os sem fins lucrativos 31%, e os empresariais somam 9%.

A rede hospitalar brasileira também é caracterizada pela ampla predominância de hospitais gerais (81%), destinados ao atendimento de especialidades médicas básicas: clínica médica; clínica cirúrgica; clínica ginecológica-obstétrica; e clínica pediátrica ^9^. Em seguida estão os hospitais especializados (11%), que são majoritariamente de médio e grande porte (66,8%) e concentrados em municípios com mais de 100 mil habitantes (87%). Existe também uma participação significativa de unidades mistas no país (7%), que são caracterizadas por terem serviços ambulatoriais básicos e pequenas unidades de internação, sendo um arranjo institucional público originalmente implementado pelo Serviço Especial de Saúde Pública (SESP) entre as décadas de 1940 e 1960 para integrar serviços preventivos e curativos ^10^. Desse modo, quase a totalidade delas são públicas (97%) e estão concentradas na Região Nordeste (69,5%), com uma proporção significativa também na Região Norte (18,2%).

Por último, os prontos-socorros gerais e especializados somam pouco mais de 1% dos hospitais e são instituições em sua maioria públicas (88,2%). Estão concentrados em municípios com população superior a 100 mil habitantes (78,43%), sendo os gerais predominantemente de pequeno porte (75,7%) e os especializados majoritariamente de médio e grande porte (71,5%).

Para aprofundar a análise do perfil dos hospitais públicos e privados que atendem ao SUS, a Tabela 1 demonstra um conjunto de indicadores hospitalares em termos de média, desvio padrão e resultados estatísticos da análise de variância, que evidenciou diferenças significativas em relação às três naturezas jurídicas.

A interpretação estatística dos indicadores hospitalares fornece um vislumbre das diferenças operacionais entre hospitais públicos, hospitais privados sem fins lucrativos e hospitais privados empresariais, refletido nas diferenças do volume e custos das internações, no perfil de atendimento, e na qualidade e na eficiência dos serviços prestados.

Nos indicadores de produção, o volume de internações se destaca por ser mais alto em hospitais sem fins lucrativos (271,46) em comparação com hospitais públicos (210,45) e empresariais (174,99). A análise estatística confirma a significância estatística dessas diferenças (Welch’s ANOVA: F = 6,49, p < 0,0001), exceto entre hospitais públicos e empresariais de acordo com os valores do teste de Tukey-Kramer. Já os valores por internação são substancialmente maiores em hospitais empresariais (R$ 2.204,47), sendo o dobro que nos sem fins lucrativos (R$ 1.020,54) e cerca de quatro vezes maior do que nos públicos (R$ 615,88), com diferenças significativas (F = 77,44, p < 0,0001). Esse padrão se repete no valor por dia de internação, com hospitais empresariais tendo um valor cerca de três vezes maior (R$ 487,77) em comparação com os hospitais públicos (R$ 115,33) e os sem fins lucrativos (R$ 191,00). Para ambos os valores, o teste de Tukey-Kramer evidencia que as diferenças são significativas entre todas as naturezas jurídicas.

Analisando os indicadores de perfil de atendimento, as internações por condições sensíveis à atenção primária (ICSAP) são ligeiramente maiores em hospitais públicos (31,4%) do que em empresariais (25,4%) e sem fins lucrativos (26,1%), com diferenças significativas apenas dos hospitais públicos em relação aos privados. Já as internações de alta complexidade são maiores nos hospitais privados, com 15,1% nos empresariais e 5% nos sem fins lucrativos, e representam somente 1,4% das internações nos hospitais públicos. Essas diferenças são muito significativas (F = 81,35, p < 0,0001) e existem entre todas as naturezas jurídicas. Em relação às internações de urgência, os hospitais públicos apresentam o maior percentual (85%), seguidos dos sem-fins lucrativos (79,8%) e, por último, dos empresariais (72,7%), com diferenças significativas entre os três grupos de hospitais. Em relação às internações de pacientes que não residem no mesmo município do hospital, as taxas são maiores nos hospitais empresariais (34,6%), com os sem fins lucrativos apresentando 26,3% e os públicos apenas 18,3%, todos diferindo estatisticamente entre si.

Em relação aos indicadores de qualidade, a taxa de mortalidade hospitalar só é significativamente maior nos hospitais sem fins lucrativos (4%) em relação aos hospitais públicos (3,1%) e empresariais (2,8%). Já a taxa de transferência hospitalar apresenta diferenças significativas entre todas as naturezas jurídicas (F = 46,37, p < 0,0001), sendo maior nos hospitais públicos (5,7%), intermediária nos sem fins lucrativos (4,1%) e menor nos empresariais (2,9%).

Quanto aos indicadores de performance, o tempo médio de permanência apresentou uma variação mais discreta (F = 4.75, p = 0,0088) e que só é estatisticamente significativa menor nos hospitais públicos (5,6 dias) quando em relação aos sem-fins lucrativos (6 dias) e empresariais (6,2 dias). Já a taxa de ocupação hospitalar é mais elevada em hospitais sem fins lucrativos (54%), sendo estatisticamente significativa (F = 61,48, p < 0,0001) em relação aos hospitais públicos (43,7%) e empresariais (43%), que quase não diferem entre si. Por último, o índice de rotatividade também é significativamente maior somente nos hospitais sem fins lucrativos (3) em relação aos hospitais empresariais (2,6) e públicos (2,3).

Os resultados da análise de variância de Welch indicaram diferenças estatisticamente significativas associadas tanto à natureza jurídica quanto ao porte de leitos dos hospitais. Por meio desses resultados, conduzimos análises adicionais para discernir possíveis efeitos moderadores que poderiam influenciar na magnitude ou na direção do desempenho entre hospitais públicos e privados. Dentro do escopo da ANOVA, o efeito de moderação é caracterizado pela interação entre a variável independente e a variável moderadora, com foco na variável dependente. Desse modo, a análise de moderação foi utilizada para explorar a relação entre o número de leitos hospitalares (variável independente) e três indicadores-chave de desempenho hospitalar: média de permanência; índice de rotatividade e taxa de ocupação (variáveis dependentes), considerando a natureza jurídica dos hospitais como variável moderadora. Essa abordagem permitiu uma compreensão mais profunda de como diferentes tipos de natureza jurídica (públicas, sem fins lucrativos, empresariais) influenciam essas relações.

Para a média de permanência, o teste de moderação demonstra efeitos significativos para as três naturezas jurídicas. Observou-se uma correlação moderada entre o número de leitos e a média de permanência em hospitais públicos, indicada por um coeficiente de correlação (R) de 0,347. A variação explicada por essa relação (R^2^) foi de 12%, com um coeficiente de interação de 0,016. Isso sugere que, embora haja alguma relação entre o número de leitos e a média de permanência em hospitais públicos, ela não é fortemente afetada pela natureza jurídica. Em hospitais sem fins lucrativos, a correlação foi mais baixa (R = 0,223) e a variação explicada ainda menor (5%), com um coeficiente de interação de 0,011. Isso indica uma influência menos significativa do número de leitos sobre a média de permanência nesse tipo de hospital. Para hospitais empresariais, a correlação foi moderada (R = 0,312), com aproximadamente 10% da variação explicada (R^2^ = 0,097) e um coeficiente de interação de 0,021, indicando uma influência moderada do número de leitos sobre a média de permanência.

**Figura 1:**
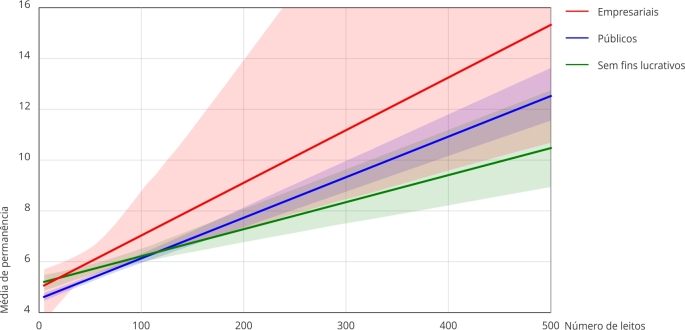
Sumário da moderação e gráfico da natureza jurídica em relação ao tempo médio de permanência e número de leitos.

Esses resultados demonstram que o aumento no tempo médio de permanência em função do número de leitos é sensivelmente maior nos hospitais públicos e empresariais. Já nos hospitais sem fins lucrativos, há uma maior estabilidade do indicador nos diferentes portes hospitalares. Esses resultados são demonstrados na Figura 1.

Nota: Hospitais públicos (R = 0,347; R^2^ = 0,120; Coeficiente de interação = 0,016; p < 0,0001; R^2^-change = 0,120; Efeitos condicionais = 4,54; p < 0,0001); Hospitais privados sem fins lucrativos (R = 0,223; R^2^ = 0,050; Coeficiente de interação = 0,011; p < 0,0001; R^2^- change = 0,049; Efeitos condicionais = 5,15; p < 0,0001); Hospitais privados empresariais (R = 0,312; R^2^ = 0,097; Coeficiente de interação = 0,021; p < 0,0001; R^2^- change = 0,095; Efeitos condicionais = 4,96; p < 0,0001).

**Figura 2:**
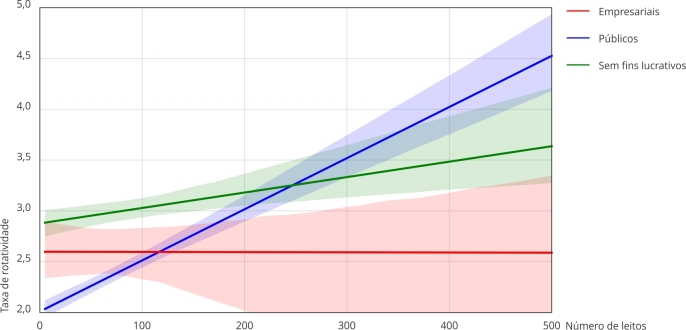
Sumário da moderação e gráfico da natureza jurídica em relação à taxa de rotatividade de leitos e número de leitos.

No que diz respeito ao índice de rotatividade, o teste de moderação mostra uma relação significativa entre a taxa de rotatividade e a natureza jurídica, mas com um perfil muito diferente para as três classes de hospitais. A análise revelou uma correlação baixa a moderada em hospitais públicos (R = 0,229), com apenas 5% da variação explicada (R^2^ = 0,053) e um coeficiente de interação de 0,005. Essa descoberta sugere que o número de leitos tem um impacto moderado sobre o índice de rotatividade em hospitais públicos. Em contraste, a relação foi muito mais fraca em hospitais sem fins lucrativos (R = 0,074) e praticamente inexistente em hospitais empresariais (R = 0,001), com R^2^ e coeficientes de interação próximos de zero. Esses resultados (Figura 2) indicam que a natureza jurídica tem um papel maior na modulação da relação entre o número de leitos e o índice de rotatividade para hospitais públicos. Para os privados, resulta em uma taxa mais equilibrada em diferentes portes hospitalares.

Nota: Hospitais públicos (R = 0,229; R^2^ = 0,053; Coeficiente de interação = 0,005; p < 0,0001; R^2^-change = 0,052; Efeitos condicionais = 2,01; p < 0,0001); Hospitais privados sem fins lucrativos (R = 0,074; R^2^ = 0,005; Coeficiente de interação = 0,002; p = 0,0032; R^2^-change = 0,005; Efeitos condicionais = 2,88; p < 0,0001); Hospitais privados empresariais (R = 0,001; R^2^ = 0,000; Coeficiente de interação = -0,000; p = 0,988; R^2^-change = -0,003; Efeitos condicionais = 2,60; p < 0,0001).

**Figura 3:**
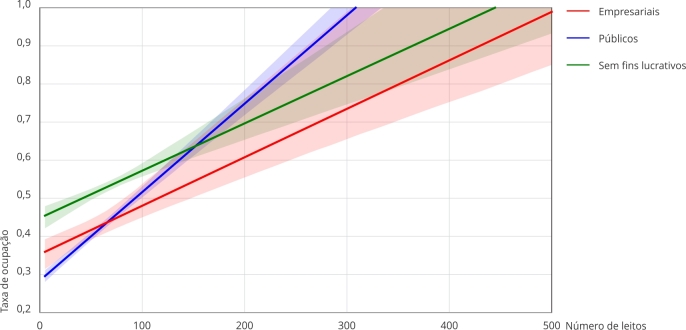
Sumário e gráfico da moderação da natureza jurídica em relação à taxa de ocupação e número de leitos.

Por fim, o teste de efeito moderador realizado para o indicador de taxa de ocupação em função da categoria de natureza jurídica foi significativo para as três classes de hospitais. A análise demonstrou uma forte correlação com o número de leitos em hospitais públicos (R = 0,572), com 33% da variação explicada (R^2^ = 0,327) e um coeficiente de interação de 0,0023. Esse resultado sugere que, em hospitais públicos, a capacidade, medida pelo número de leitos, é um fator importante na determinação da taxa de ocupação. Uma relação similar, embora moderada, foi observada em hospitais sem fins lucrativos (R = 0,406, R^2^ = 0,165) e empresariais (R = 0,406, R^2^ = 0,165), com coeficientes de interação de 0,0012 e 0,0013, respectivamente. Esses achados indicam que, embora a taxa de ocupação também seja influenciada pelo número de leitos nesses hospitais, a natureza jurídica desempenha um papel menos proeminente em comparação aos hospitais públicos, que partem das menores taxas nos hospitais menores, mas possuem maiores taxas nos maiores portes hospitalares. Esses resultados são demonstrados na Figura 3.

Nota: Hospitais públicos (R = 0,572; R^2^ = 0,327; Coeficiente de interação = 0,002; p < 0,0001; R^2^-change = 0,327; Efeitos condicionais = 0,28; p < 0,0001); Hospitais privados sem fins lucrativos (R = 0,406; R^2^ = 0,165; Coeficiente de interação = 0,001; p < 0,0001; R^2^-change = 0,165; Efeitos condicionais = 0,45; p < 0,0001); Hospitais privados empresariais (R = 0,406; R^2^ = 0,165; Coeficiente de interação = 0,001; p < 0,0001; R^2^-change = 0,162; Efeitos condicionais = 0,35; p < 0,0001).

**Figura 4:**
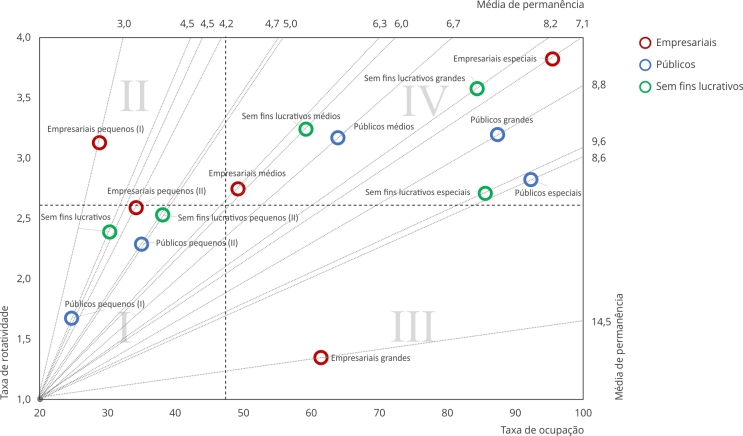
Modelo Pabon Lasso dos hospitais por porte e natureza jurídica.

No estudo sobre desempenho hospitalar, o modelo Pabon Lasso foi empregado para uma análise integrada. Esse modelo utiliza um gráfico de coordenadas cartesianas, com a taxa de ocupação no eixo das abscissas, o índice de rotatividade no eixo das ordenadas e linhas de média de permanência interseccionando essas variáveis, formando quatro quadrantes ^5^. Cada um deles no modelo Pabon Lasso representa um nível de desempenho: o quadrante inferior esquerdo (I) indica baixo desempenho devido a baixas taxas de ocupação e rotatividade; o superior direito (IV) mostra alto desempenho com boa ocupação de leitos e alta produtividade; enquanto os quadrantes II e III representam desempenhos intermediários, com o II indicando alta produtividade, mas subutilização de recursos, e o III alta ocupação de leitos, mas menor produtividade em termos de internações ^5^. Utilizando esse método, foi possível criar um recurso visual claro, sintetizando os resultados do estudo e proporcionando uma visão integrada do desempenho dos hospitais brasileiros, agrupados por natureza jurídica e porte hospitalar como demonstrado na Figura 4.

Os resultados da aplicação do modelo Pabon Lasso demonstram que, nos hospitais de porte especial, tanto os empresariais (taxa de ocupação = 95,5% e índice de rotatividade = 3,8), quanto os públicos (taxa de ocupação = 92,2% e índice de rotatividade = 2,8) e sem fins lucrativos (taxa de ocupação = 85,6% e índice de rotatividade = 2,7) se posicionam no quadrante IV, com variações discretas. Essa localização indica uma gestão operacional eficiente, com alta ocupação e rotatividade de leitos.

Em relação aos hospitais de grande porte, os sem fins lucrativos (com uma taxa de ocupação de 84,3% e índice de rotatividade de 3,8) e os públicos (com uma taxa de ocupação de 87,4% e índice de rotatividae de 3,2) também se encontram no quadrante IV, mostrando alta performance. Mas os hospitais empresariais grandes, com uma taxa de ocupação mais baixa de 61,4% e uma índice de rotatividade de 1,3, se localizam no quadrante III, o que é indicativo de uma performance intermediária com ocupação menor e rotatividade mais baixa.

Nos hospitais de porte médio, a situação é mista. Os hospitais empresariais, com uma taxa de ocupação de 49,1% e índice de rotatividade de 2,7, assim como os públicos e sem fins lucrativos, com taxa de ocupação de 63,8% e 59,1%, respectivamente, e índice de rotatividade de 3,2, tendem a se situar entre os quadrantes II e III, representando uma performance intermediária.

Para os hospitais de pequeno porte II, a tendência é de menor eficiência, com todos os hospitais posicionados no quadrante I, o que indica baixa performance. Finalmente, nos hospitais pequenos I, essa tendência de baixa performance é mais acentuada para públicos e sem fins lucrativos posicionados no quadrante I, com destaque de menor performance dos hospitais públicos (taxa de ocupação = 24,7% e índice de rotatividade = 1,7). Somente os empresariais apresentam uma performance intermediária com taxa de ocupação de 28,7% e índice de rotatividade de 3,2, estando localizados no quadrante II.

## Discussão

A análise inicial dos dados revelou que um alto percentual de hospitais (15,2%) foi excluído do estudo devido à ausência de registros de internações, apesar de possuírem no CNES todas as características cadastrais necessárias para a realização de internações pelo SUS. Apesar de ser um fato preocupante, relatos sobre problemas de qualidade dos dados do SIH são bem documentados por estudos anteriores. Fenômenos como a subnotificação de AIH, a baixa qualidade dos diagnósticos e a manipulação da codificação de procedimentos visando a maximização do faturamento são temas recorrentes nos estudos sobre o sistema, que normalmente associam esses e outros problemas como inerentes ao modelo de reembolso de pagamento por serviços prestados (*fee-for-service*) utilizado pelo SUS para remunerar seus prestadores de serviço ^11^ ^,^ ^12^ ^,^ ^13^ ^,^ ^14^ ^,^ ^15^ ^,^ ^16^ ^,^ ^17^. Diante da solidez desses achados prévios, é imprescindível reconhecer a ausência de registros por subnotificação de AIH como uma limitação substancial dos dados empregados nesta pesquisa. Essa constatação sublinha a urgência de estratégias mais eficazes para a coleta e a análise de dados no contexto hospitalar do SUS, visando à melhoria contínua na precisão das informações geradas.

Assim como demonstrado por Ugá & López ^18^, este estudo confirmou que a maior parte dos hospitais brasileiros (61%) se caracteriza por seu pequeno porte e ampla difusão em território nacional. Esse fenômeno pode ser explicado pela estratégia adotada pelo SUS para promover um modelo de organização de atendimento em que hospitais de pequeno porte desempenham um papel estratégico na composição das Redes de Atenção à Saúde ^19^, que inclusive é incentivado por meio de mecanismos de financiamento específicos ^20^. Ugá & López ^18^ destacam que, apesar de constituírem uma parcela majoritária da rede hospitalar brasileira, esses hospitais enfrentam limitações em complexidade e densidade tecnológica, com taxas de ocupação reduzidas e baixa resolubilidade, uma consequência de políticas insuficientes de investimento e recursos humanos. Carpanez & Malik ^21^ observam que a municipalização do sistema hospitalar contribuiu para o crescimento do número de hospitais de pequeno porte, mas essa expansão não foi acompanhada de melhorias na qualidade e na integração do atendimento, resultando em um sistema hospitalar fragmentado.

O estudo demonstra um aumento substancial na eficiência de escala relacionada com o aumento de leitos, corroborando as conclusões de pesquisas anteriores ^3^ ^,^ ^4^ ^,^ ^22^. Observou-se que, à medida que o número de leitos aumenta, há uma melhoria significativa nos indicadores, evidenciando essa relação entre a capacidade hospitalar ampliada e a otimização do desempenho. Assim, como em Ramos et al. ^3^, foi identificado que hospitais maiores apresentam maior taxa de ocupação e índice de rotatividade de leitos, indicando uma maior eficiência operacional. Da mesma forma, como para Botega et al. ^4^, o tamanho do hospital é a variável que mais influenciou nos indicadores, com os grandes oferecendo mais cuidados de alta complexidade, apresentando uma maior taxa de ocupação e maior cobertura geográfica, o que demonstra a importância da escala de produção na definição do perfil e do desempenho dos hospitais.

Essa relação entre o volume de procedimentos médicos e a qualidade dos resultados é complexa e tem sido amplamente estudada sob o prisma da obra de Donabedian ^23^, que avalia a qualidade do cuidado em saúde por meio de três pilares: estruturas, processos e resultados. Luft et al. ^24^ introduziram a discussão sobre a “relação volume-resultado”, apresentando a hipótese de que a prática frequente de procedimentos pode levar ao aperfeiçoamento das habilidades (“a prática leva à perfeição”) e a hipótese de que pacientes tendem a ser encaminhados para prestadores com melhores resultados (“encaminhamento seletivo”). Noronha et al. ^25^ corroboraram essa relação no contexto da cirurgia coronariana no Brasil, indicando que um maior volume de procedimentos está associado a melhores resultados. Essas pesquisas coletivamente sugerem que tanto a experiência acumulada quanto os padrões de encaminhamento influenciam a qualidade do cuidado em saúde.

Se essa relação entre porte hospitalar (ou volume de produção) com os resultados em saúde já é consolidada na literatura nacional e internacional e apontam para resultados similares, estudos de desempenho que envolvem hospitais públicos e privados são menos frequentes e apresentam resultados mais diversos. O estudo de Martins et al. ^26^, em São Paulo, encontrou um melhor desempenho clínico dos hospitais públicos em termos de mortalidade hospitalar ajustada, mas não foram encontradas diferenças estatísticas significativas em relação ao tempo médio de permanência das internações. Utilizando uma metodologia qualitativa e quantitativa para analisar hospitais de São Paulo, Rotta ^27^ conclui que hospitais sem fins lucrativos e públicos apresentaram menor produtividade em indicadores de utilização de leitos e produtividade do centro cirúrgico do que os empresariais. Já Santana ^28^, ao utilizar análise envoltória de dados para analisar 27 hospitais de oito estados brasileiros, concluiu que os hospitais públicos foram os que obtiveram melhor desempenho. No entanto, neste estudo, encontramos variações significativas em diversos indicadores para as diferentes naturezas jurídicas.

Em termos de custos de internação e complexidade dos casos tratados, os hospitais empresariais se destacam com valores mais elevados, refletindo o tratamento de casos clínicos mais complexos e o uso de tecnologias de alto custo.

No que tange ao perfil de atendimentos, os hospitais públicos registram um número maior de ICSAP, indicador de qualidade da assistência que avalia tanto a efetividade das ações da atenção primária, quanto a organização geral dos serviços de saúde ^29^. Em contrapartida, os hospitais empresariais concentram um maior volume de internações de alta complexidade, evidenciando uma grande disponibilidade de serviços especializados e recursos avançados. Além disso, as internações de emergência são mais frequentes nos hospitais públicos, enquanto os empresariais atraem uma proporção maior de pacientes de outros municípios, refletindo sua especialização e capacidade de atender casos mais complexos.

No que se refere à qualidade dos serviços, os hospitais sem fins lucrativos apresentam taxas mais elevadas de mortalidade hospitalar, o que não indica necessariamente problemas na qualidade do atendimento, pois a variação nessa taxa pode ser atribuída a uma série de fatores, incluindo a gravidade do estado de saúde da população atendida, a eficácia das tecnologias médicas empregadas, a adequação do processo de cuidado ao paciente e os erros aleatórios ^30^. Por outro lado, os hospitais públicos apresentam taxas maiores de transferência hospitalar, sugerindo baixa resolubilidade e falta da infraestrutura necessária para tratar casos clínicos mais complexos internamente ^3^.

Nos indicadores de desempenho, os hospitais empresariais demonstraram um tempo médio de permanência significativamente mais elevado quando comparado aos públicos, mas com uma taxa de crescimento por número de leitos muito similar entre eles, enquanto os sem fins lucrativos apresentam uma taxa intermediária e mais equilibrada com o aumento de leitos. O tempo médio de permanência é um indicador relacionado a boas práticas clínicas, gestão eficiente de leitos e rotatividade operacional de leitos. Somente médias superiores a sete dias estão associadas ao aumento do risco de infecção hospitalar ^31^, patamar que não é atingido pela maioria dos hospitais brasileiros, mas que hospitais de porte grande e especial de todas as naturezas jurídicas ultrapassam.

Já o índice de rotatividade, que mensura o número médio de internações por leito em um mês, apresentou variação entre todas as naturezas jurídicas, com taxas médias mais altas em hospitais privados sem fins lucrativos e mais baixas em hospitais públicos. Entretanto, os hospitais públicos apresentam um crescimento acentuado desse índice com o aumento do número de leitos, demonstrando ganhos de eficiência de escala e taxas maiores do que os hospitais privados nos maiores portes. É um indicador importante de produtividade e desempenho, mas valores altos também podem indicar reinternações, internações desnecessárias ou altas precoces ^3^.

Por último, a taxa de ocupação de leitos se apresentou maior em hospitais privados sem fins lucrativos, com os hospitais públicos e empresariais sem diferenças significativas entre si. Entretanto, os hospitais públicos apresentam o maior crescimento desse indicador com o crescimento dos leitos, demonstrando novamente ganhos de eficiência de escala e taxas maiores do que os hospitais privados nos maiores portes. Esse indicador está diretamente relacionado à gestão eficiente de leitos, pois resulta em uma maior oferta para o sistema de saúde. Estima-se que a ocupação ideal de leitos esteja entre 75% e 85%, com taxas abaixo desse parâmetro indicando baixa utilização e ineficiência na gestão hospitalar, e taxas mais altas estando relacionadas a um aumento em eventos adversos, infecção hospitalar e/ou diminuição da segurança no ambiente de atendimento ^31^. Nesse aspecto, é importante destacar que, na média, todos os hospitais brasileiros estão muito abaixo desse parâmetro independentemente da natureza jurídica, fato diretamente relacionado com a predominância de hospitais de pequeno porte e suas baixas taxas de ocupação. No outro extremo, as taxas de ocupação de hospitais grandes e especializados variam entre 86,5% e 91,1%, ou seja, taxas acima do ideal para esse indicador.

## Conclusão

A análise de moderação evidenciou que a natureza jurídica exerce um efeito moderador na relação entre os três indicadores de desempenho e o porte do hospital. Esse efeito demonstra que os hospitais públicos apresentam o maior crescimento dos três indicadores de desempenho em relação ao porte hospitalar, resultando em maiores ganhos em eficiência de escala como resultado do maior volume de internações. Por outro lado, os hospitais privados sem fins lucrativos apresentam um melhor desempenho no conjunto dos três indicadores, que apresentam uma variação mais moderada nos diferentes portes hospitalares.

A aplicação do modelo Pabon Lasso confirmou as descobertas da análise de moderação e ofereceu uma visão integrada do desempenho dos hospitais brasileiros. Os resultados apontam que hospitais pequenos, tanto públicos quanto privados, apresentam baixo desempenho, e que a partir do médio porte, os hospitais públicos e sem fins lucrativos atingem boa performance e são muito similares. Já os hospitais empresariais de médio e grande porte apresentaram um desempenho intermediário, tendo os de médio porte com baixa taxa de ocupação de leitos e os de grande porte uma baixa produção de internações por leito. Os hospitais empresariais especiais foram os que apresentaram um desempenho geral mais elevado no conjunto de indicadores.

Desse modo, os resultados do estudo evidenciaram que o desempenho hospitalar tende a melhorar com o aumento do número de leitos para todos os hospitais brasileiros, reforçando estudos prévios realizados em contextos mais restritos. No entanto, também foi demonstrado que o perfil de desempenho varia significativamente em função da natureza jurídica das instituições hospitalares. Nessa perspectiva, os hospitais públicos demonstram um aumento de desempenho mais acentuado com a expansão de seu porte, evidenciando maiores ganhos de eficiência de escala. Já os hospitais privados sem fins lucrativos, apesar de apresentaram a mesma tendência, mantêm um desempenho mais elevado e com menos variações nos diferentes portes. Os hospitais empresariais apresentaram maiores variações em seu desempenho, com os de grande porte apresentando resultados intermediários e os especiais demonstrando um desempenho geral mais elevado.

## References

[1] Mendes EV (2011). As redes de atenção à saúde.

[2] Schout D, Novaes HM (2007). Do registro ao indicador gestão da produção da informação
assistencial nos hospitais. Ciênc Saúde Colet.

[3] Ramos MC, Cruz LP, Kishima VC, Pollara WM, Lira AC, Couttolenc BF (2015). Avaliação de desempenho de hospitais que prestam atendimento pelo
sistema público de saúde, Brasil. Rev Saúde Pública.

[4] Botega LD, Andrade MV, Guedes GR (2020). Perfil dos hospitais gerais do Sistema Único de
Saúde. Rev Saúde Pública.

[5] Pabón Lasso H (1984). Método simplificado para evaluar el desempeño
hospitalario. Bol Oficina Sanit Panam.

[6] Paim J, Travassos C, Almeida C, Bahia L, Macinko J (2011). The Brazilian health system history, advances, and
challenges. Lancet.

[7] Ferrarini CD (1977). Conceitos e definições em saúde. Rev Bras Enferm.

[8] Baron RM, Kenny DA (1986). The moderator-mediator variable distinction in social
psychological research conceptual, strategic, and statistical
considerations. J Pers Soc Psychol.

[9] Ministério da Saúde (2002). Portaria nº 356, de 20 de fevereiro de 2002. Aprova o Glossário
de Termos Comuns nos Serviços de Saúde do MERCOSUL.. Diário Oficial da União.

[10] Mello GA, Viana AL (2012). Uma história de conceitos na saúde pública integralidade,
coordenação, descentralização, regionalização e
universalidade. Hist Ciênc Saúde-Manguinhos.

[11] Veras CM, Martins MS (1994). A confiabilidade dos dados nos formulários de Autorização de
Internação Hospitalar (AIH), Rio de Janeiro, Brasil. Cad Saúde Pública.

[12] Portela MC, Schramm JM, Pepe VL, Noronha MF, Pinto CA, Cianeli MP (1997). Algoritmo para a composição de dados por internação a partir do
sistema de informações hospitalares do sistema único de saúde (SIH/SUS) -
Composição de dados por internação a partir do SIH/SUS. Cad Saúde Pública.

[13] Melo EC, Travassos C, Carvalho MS (2004). Qualidade dos dados sobre óbitos por infarto agudo do miocárdio,
Rio de Janeiro. Rev Saúde Pública.

[14] Scatena JH, Tanaka OY (2001). Utilização do Sistema de Informações Hospitalares (SIH-SUS) e do
Sistema de Informações Ambulatoriais (SIA-SUS) na análise da
descentralização da saúde em Mato Grosso. Inf Epidemiol SUS.

[15] Nakamura-Pereira M, Mendes-Silva W, Dias MA, Reichenheim ME, Lobato G (2013). Sistema de Informações Hospitalares do Sistema Único de Saúde
(SIH-SUS) uma avaliação do seu desempenho para a identificação do near miss
materno. Cad Saúde Pública.

[16] Orlandi DP, Coelho TP, Almeida JEF Sistema de informações hospitalares (SIH-SUS): revisão sobre qualidade
da informação e utilização do banco de dados em pesquisas. In: IX Congresso
CONSAD de Gestão Pública..

[17] Bittencourt SA, Camacho LA, Leal MC (2006). O Sistema de Informação Hospitalar e sua aplicação na saúde
coletiva. Cad Saúde Pública.

[18] Ugá MA, López EM (2007). Os hospitais de pequeno porte e sua inserção no
SUS. Ciênc Saúde Coletiva.

[19] Souza FE, Nunes ED, Carvalho BG, Mendonça FD, Lazarini FM (2019). Atuação dos hospitais de pequeno porte de pequenos municípios nas
redes de atenção à saúde. Saúde Soc.

[20] Ministério da Saúde (2004). Portaria GM nº 1.044, de 01 de junho de 2004. Institui a Política
Nacional para os Hospitais de Pequeno Porte.. Diário Oficial da União.

[21] Carpanez LR, Malik AM (2021). O efeito da municipalização no sistema hospitalar brasileiro os
hospitais de pequeno porte. Ciênc Saúde Colet.

[22] La Forgia GM, Couttolenc B (2008). Hospital performance in Brazil: the search for excellence.

[23] Donabedian A (1988). The quality of care how can it be assessed?. JAMA.

[24] Luft HS, Hunt SS, Maerki SC (1987). The volume-outcome relationship practice-makes-perfect or
selective-referral patterns?. Health Serv Res.

[25] Noronha JC, Travassos C, Martins M, Campos MR, Maia P, Panezzuti R (2003). Avaliação da relação entre volume de procedimentos e a qualidade
do cuidado o caso de cirurgia coronariana no Brasil. Cad Saúde Pública.

[26] Martins M, Blais R, Leite IC (2004). Mortalidade hospitalar e tempo de permanência comparação entre
hospitais públicos e privados na região de Ribeirão Preto, São Paulo,
Brasil. Cad Saúde Pública.

[27] Rotta SG (2004). Utilização de indicadores de desempenho hospitalar como instrumento
gerencial.

[28] Santana DR (2020). Análise de desempenho de organizações hospitalares por meio de
indicadores financeiros do ano de 2017.

[29] Malvezzi E (2018). Internações por condições sensíveis a atenção primária revisão
qualitativa da literatura científica brasileira. Saúde Redes.

[30] Travassos C, Noronha JC, Martins M (1999). Mortalidade hospitalar como indicador de qualidade uma
revisão. Ciênc Saúde Colet.

[31] Agência Nacional de Saúde Suplementar Monitoramento da qualidade dos prestadores de serviços de
saúde..

[32] Cherubin NA, Santos NA (1997). Administração hospitalar, fundamentos.

[33] Projeto de Avaliação do Desempenho do Sistema de Saúde Hospitais por porte e tipo de atendimento..

